# Weighted gene co-expression network analysis of expression data of monozygotic twins identifies specific modules and hub genes related to BMI

**DOI:** 10.1186/s12864-017-4257-6

**Published:** 2017-11-13

**Authors:** Weijing Wang, Wenjie Jiang, Lin Hou, Haiping Duan, Yili Wu, Chunsheng Xu, Qihua Tan, Shuxia Li, Dongfeng Zhang

**Affiliations:** 1 Department of Epidemiology and Health Statistics, Public Health College, Qingdao University, No. 38 Dengzhou Road, Shibei District, Qingdao, 266021 Shandong Province People’s Republic of China; 2 Department of Biochemistry, Medical College, Qingdao University, No. 38 Dengzhou Road, Shibei District, Qingdao, 266021 Shandong Province People’s Republic of China; 3 Qingdao Municipal Center for Disease Control and Prevention, No. 175 Shandong Road, Shibei District, Qingdao, 266033 Shandong Province People’s Republic of China; 4 Qingdao Institute of Preventive Medicine, No. 175 Shandong Road, Shibei District, Qingdao, 266033 Shandong Province People’s Republic of China; 5 Epidemiology, Biostatistics and Bio-demography, Institute of Public Health, University of Southern Denmark, DK-5000 Odense C, Denmark; 6 Human Genetics, Institute of Clinical Research, University of Southern Denmark, DK-5000 Odense C, Denmark

**Keywords:** BMI, Differentially expressed genes, Gene module, Hub gene, Monozygotic twins, Obesity, WGCNA

## Abstract

**Background:**

The therapeutic management of obesity is challenging, hence further elucidating the underlying mechanisms of obesity development and identifying new diagnostic biomarkers and therapeutic targets are urgent and necessary. Here, we performed differential gene expression analysis and weighted gene co-expression network analysis (WGCNA) to identify significant genes and specific modules related to BMI based on gene expression profile data of 7 discordant monozygotic twins.

**Results:**

In the differential gene expression analysis, it appeared that 32 differentially expressed genes (DEGs) were with a trend of up-regulation in twins with higher BMI when compared to their siblings. Categories of positive regulation of nitric-oxide synthase biosynthetic process, positive regulation of NF-kappa B import into nucleus, and peroxidase activity were significantly enriched within GO database and NF-kappa B signaling pathway within KEGG database. DEGs of *NAMPT*, *TLR9*, *PTGS2*, *HBD*, and *PCSK1N* might be associated with obesity. In the WGCNA, among the total 20 distinct co-expression modules identified, coral1 module (68 genes) had the strongest positive correlation with BMI (*r* = 0.56, *P* = 0.04) and disease status (r = 0.56, *P* = 0.04). Categories of positive regulation of phospholipase activity, high-density lipoprotein particle clearance, chylomicron remnant clearance, reverse cholesterol transport, intermediate-density lipoprotein particle, chylomicron, low-density lipoprotein particle, very-low-density lipoprotein particle, voltage-gated potassium channel complex, cholesterol transporter activity, and neuropeptide hormone activity were significantly enriched within GO database for this module. And alcoholism and cell adhesion molecules pathways were significantly enriched within KEGG database. Several hub genes, such as *GAL*, *ASB9*, *NPPB*, *TBX2*, *IL17C*, *APOE*, *ABCG4*, and *APOC2* were also identified. The module eigengene of saddlebrown module (212 genes) was also significantly correlated with BMI (*r* = 0.56, *P* = 0.04), and hub genes of *KCNN1* and *AQP10* were differentially expressed.

**Conclusion:**

We identified significant genes and specific modules potentially related to BMI based on the gene expression profile data of monozygotic twins. The findings may help further elucidate the underlying mechanisms of obesity development and provide novel insights to research potential gene biomarkers and signaling pathways for obesity treatment. Further analysis and validation of the findings reported here are important and necessary when more sample size is acquired.

**Electronic supplementary material:**

The online version of this article (10.1186/s12864-017-4257-6) contains supplementary material, which is available to authorized users.

## Background

Obesity, as a complex disorder mediated by the interplay between genetic and environmental factors [1], has been a public health and policy problem due to its prevalence, costs, and health effects [2]. The therapeutic management of obesity includes lifestyle changes, medications, and surgery. However, the treatment of obesity is challenging because of diverse patient conditions, prolonged and chronic nature of disease, difficulty of maintaining dieting and physical exercise frequently [3–5], limited effectiveness and side effects of the medication [6], and high cost and risk of complications of surgery [7]. Other efforts are focused in the development of novel therapeutics, yet the effectiveness requires to be tested and confirmed [8–10] and the safety requires to be assessed [11]. Therefore, for the purpose of identifying new diagnostic biomarkers and therapeutic targets and developing novel therapeutic strategies which not only produce sufficient weight loss but also lack side effects, further elucidating the molecular mechanisms underlying obesity development is necessary and urgent.

Recently, gene expression profiling analysis has yielded insights into the measurement of alterations in genetic expression patterns, and has facilitated the identification of differentially expressed genes (DEGs) being crucial to obesity. In a study conducted by Roque DR, et al., obesity related genes, such as *LPL, IRS-1, IGFBP4,* and *IGFBP7*, etc., were found to be upregulated with increasing BMI among endometrial cancer patients [12]. The study of Gruchala-Niedoszytko, M, et al. also found a series of genes (*PI3, LOC100008589, RPS6KA3, LOC441763, IFIT1,* and *LOC100133565*) with a different expression that may be related to an increased BMI [13]. And genes of *PGC1-*α, *FNCD5,* and *FGF*, which play roles in adipose tissue development and function, were abundantly expressed in subcutaneous, visceral, and epigastric adipose tissues of extreme obesity patients based on gene expression profiling [14]. However, due to the gene expression profiling analysis merely focused on the effect of individual genes and transcripts, without regard to their correlated patterns of expression and the effect of networks of genes, it may fail to detect important biological pathways or gene-gene interactions related to obesity.

Weighted gene co-expression network analysis (WGCNA) is a systems biology method for analyzing the correlation patterns of large and high-dimensional gene expression data sets [15]. It can be used to find modules of highly correlated genes, correlate module eigengenes (MEs) to external sample traits, calculate module membership (MM) and gene significance (GS), and find intramodular hub genes, etc. WGCNA has yielded novel insights into the molecular aspects to identify candidate biomarkers or therapeutic targets. At present, it has been increasingly applied to analyze various gene expression profiles of hepatocellular carcinoma [16], pneumocyte senescence induced by thoracic irradiation [17], psoriasis [18], severe asthma [19], coronary artery disease [20], and lung cancer [21], etc. Although widely being employed, the WGCNA has, to our knowledge, not yet been applied to analyze the expression profiles of BMI-discordant monozygotic twin pairs.

While monozygotic twins are characteristic of the genetic similarity and rearing-environment sharing, they show phenotypic discordance for certain complex traits and diseases. Thus, the discordant monozygotic model is becoming a popular and powerful tool for identifying non-genetic contributions to a phenotype variation including subtle difference in gene expression not mediated by cis- or trans-eQTL effects, and for linking environmental exposure to differential epigenetic regulation while controlling for individual genetic make-up [22–24]. Therefore, to reveal the potential molecular mechanisms of obesity, we performed both differential gene expression analysis and WGCNA to analyze the expression profiles of BMI-discordant monozygotic twin pairs. The potentially important DEGs were identified, and the modules correlated with external traits and the hub genes related to BMI were determined. The results may help further elucidate the underlying mechanisms of obesity and provide novel insights to research potential gene biomarkers and signaling pathways for the treatment of obesity.

## Methods

### Subjects recruitment

The sampling of monozygotic twins was based on the Qingdao Twin Registry at the Qingdao Center for Disease Control and Prevention [25]. Twins were recruited to a clinical investigation after sampling randomly through residence registry and the local disease control network (2012–2013). Written informed consent was obtained from all subjects. We excluded subjects (i) being pregnant or breastfeeding, (ii) undergoing diabetes, (iii) undergoing cardiovascular disease, and (iv) taking any medications within 1 month before participation, and incomplete twin pairs were dropped. The zygosity of twin pairs was determined by DNA testing using 16 short tandem repeat DNA markers. Finally, a total of 7 BMI-discordant monozygotic twin pairs with median age of 52 years (range: 43–65 years) were identified.

For each subject, we took three anthropometric measurements following standard procedures with at least one-minute interval and calculated the mean of these three measurements. Height was measured to the nearest centimeter using a vertical scale with a horizontal moving headboard. And body weight was measured to the nearest 0.1 kg using a standing beam scale. Then BMI was calculated as weight (kg) divided by the square of height (m). Besides, BMI was classified into three classes: Class I, 18.5 ≤ BMI < 24 kg/m^2^, normal; Class II, 24 ≤ BMI < 28 kg/m^2^, overweight; and Class III, BMI ≥ 28 kg/m^2^, obesity. Blood sample was kept frozen at −80 °C for 6 months before sending to routine laboratory testing.

### RNA library construction and sequencing and quality control

After total messenger RNA (mRNA) being extracted from whole blood by using TRIzol reagent (Invitrogen, San Diego, USA), the RNA concentration and purity were tested with NanoDrop 2000 Spectrophotometer (Termo Fisher Scientifc, Wilmington, USA) and the RNA integrity was measured with RNA Nano 6000 Assay Kit of Agilent Bioanalyzer 2100 system (Agilent Technologies, Santa Clara, USA).

Then the high-quality RNA was sent to Biomarker Technologies Corporation (Biomarker Technologies Corporation, Beijing, China) for further analysis. The RNA-Seq libraries were constructed with NEBNext UltraTM RNA Library Prep Kit for Illumina (New England Biolabs, Ipswich, USA) following the manufacturer’s recommendations as follows: purifying mRNA with NEBNext Poly (A) mRNA Magnetic Isolation Module, randomly fragmenting isolated mRNA, synthesizing and purifying double-stranded cDNAs, selecting fragment sizes using Agencourt AMPure XP system, and obtaining cDNA library by PCR enrichment. At last, we sequenced the prepared cDNA library using the Illumina HiSeq 2500.

To obtain high-quality clean data (Q30 > 85%), quality control for the raw sequencing data was performed by removing reads containing adapter sequences, unknown nucleotides >5%, and low-quality reads. After mapping to the human genome by TopHat2 [26], we estimated the gene expression levels with fragments per kilobase of exon per million fragments mapped (FPKM) value by Cufflinks software [27].

### Differential expression analysis

In the differential expression analysis between the 7 BMI-discordant twin pairs using EBSeq [28], the Benjamini-Hochberg method corrected *P*-value, i.e., False discovery rate (FDR), was estimated to circumvent false positive results which occurred in the multiple tests [29, 30]. The fold change (FC) of the expression values between twins was also calculated. Then DEGs were defined as those met the criteria of |log2FC| > 1and FDR < 0.01.

### Weighted gene co-expression network analysis (WGCNA)

The WGCNA package in R is a comprehensive collection of R functions for performing various aspects of weighted correlation network analysis [15, 31]. Based on the expression profiles of 7 monozygotic twin pairs, the network construction, module detection, module and gene selection, calculations of topological properties, visualization, and interfacing with external software package were conducted following the tutorials provided.

#### Modules identification

Briefly, after calculating Pearson correlations between each gene pair, we established a weighted adjacency matrix by raising the co-expression similarity to a power β = 29. Subsequently, we constructed the topological overlap matrix (TOM) using correlation expression values [32–34]. Then each TOM was used as input for hierarchical clustering analysis [35], and gene modules (i.e. clusters of genes with high topological overlap) was detected by using a dynamic tree cutting algorithm (deep split = 2, cut height = 0.27). The co-expression module structure was visualized by heatmap plots of topological overlap in the gene network. Relationships among modules were summarized by a hierarchical clustering dendrogram of the eigengenes and by a heatmap plot of the corresponding eigengene network.

#### Relating modules to external traits

To identify modules that were significantly associated with the traits of interest-BMI, BMI classes, and disease status (obesity versus non-obesity), we correlated the MEs (i.e. the first principle component of a module) [36] with external traits and searched the most significant associations.

#### Hub genes analysis

The MM was defined as the correlation of gene expression profile with ME. And the GS measure was defined as (the absolute value of) the correlation between gene and external traits. Genes with highest MM and highest GS in modules of interest were natural candidates for further research [37–40]. Thus, the intramodular hub genes were chosen by external traits based GS > 0.2 and MM > 0.8 with a threshold of *P*-value <0.05 [41]. The gene-gene interaction network was constructed and visualized using VisANT 5.0 [42].

### Functional annotation and enrichment analysis

Genes identified in the differential expression analysis and in module of interest in WGCNA were annotated by utilizing BLAST software within the following databases: NCBI nonredundant protein sequences (NR) [43], Clusters of Orthologous Groups (COG) [44], KOG [45], Kyoto Encyclopedia of Genes and Genomes (KEGG) [46, 47], and Gene Ontology (GO) [48, 49]. Subsequently, we drew histogram by mapping GO function of genes in modules of interest to the corresponding secondary features on the background of all genes’ GO annotation. The Pearson Chi-Square test was applied to indicate significant relationships between the two input datasets if all the expected counts were greater than or equal to 5 for 2 × 2 matrixes. And the Fisher’s exact test was applied if one of the expected counts was less than 5. Then we implemented GO enrichment analysis based on a hypergeometric test [50] and calculated a Fisher’s Exact *P*-Value which was then corrected by Benjamini-Hochberg method. Besides, the KEGG pathways enrichment analysis was conducted by applying the KEGG Orthology-Based Annotation System (KOBAS) utilizing a hypergeometric test [51]. The *P*-value <0.05 was used as the enrichment cut-off criterion.

## Results

### Differential expression analysis

A total of 7 BMI-discordant monozygotic twin pairs with median age of 52 years were included for the gene expression profiling analysis (Table 1). The extracted cDNA samples from twins were subjected to sequencing using an Illumina HiSeq2500 platform. The Q30 of each sample was not less than 92.26% and the mapped rate ranged from 87.62% to 93.18% (Additional file 1: Table S1). Under the threshold of |log2FC| > 1 and FDR < 0.01, a range from 360 to 1116 DEGs were identified between co-twin pairs (Table 1). It appeared that 32 DEGs were with a trend of up-regulation in at least three of twins with higher BMI when compared to their siblings (Additional file 2: Table S2). Of these, three genes were found up-regulated in 4 twin pairs, and the others were found up-regulated in 3 twin pairs.

**Table 1:** The characteristics of the BMI-discordant monozygotic twin pairs (43–65 years) and summary of differentially expressed genes

Subject ID	Height, m	weight, kg	BMI, kg/m^2^	DEG Set	All DEGs	Up-regulated	Down-regulated
E01	1.56	72	29.6				
E02	1.54	62	26.1	E02_vs_E01	462	418	44
E03	1.62	89	34.1				
E04	1.63	73	27.5	E04_vs_E03	1116	579	537
E05	1.65	53	19.5				
E06	1.6	65.3	25.5	E05_vs_E06	656	356	300
E07	1.73	67.7	22.6				
E08	1.72	75.9	25.7	E07_vs_E08	576	426	150
E09	1.67	81.4	29.2				
E10	1.66	67.2	24.5	E10_vs_E09	360	163	197
E11	1.7	55.9	19.3				
E12	1.7	71.2	24.6	E11_vs_E12	625	187	438
E13	1.55	63	26.2				
E14	1.56	71	29.2	E13_vs_E14	661	426	235

Note: DEG: differentially expressed gene; Up-regulated: the number of up-regulated genes; Down-regulated: the number of down-regulated genes

As the summarized results of enrichment analysis within GO and KEGG databases shown (Table 2), several potentially important findings emerged (Corrected *P*-value < 0.05), including positive regulation of nitric-oxide synthase biosynthetic process (*P* = 5.34E-03), positive regulation of NF-kappa B import into nucleus (*P* = 1.04E-02), peroxidase activity (*P* = 6.82E-03), and NF-kappa B signaling pathway (*P* = 4.49E-02). Genes of *NAMPT*, *TLR9*, *PTGS2*, *HBD*, and *BCL2L1* were involved in these significant findings. In addition, *PCSK1N* gene might also be associated with obesity. We compared previously implicated BMI-related gene expression differences in study of Homuth, G, et al. [52] with ours to validate the findings further. This comparison revealed consistency for positive BMI-associated expression differences, including *HBD*, *XK*, *SELENBP1*, *SNCA*, *LAS2*, *PLEK2*, *GLRX5*, *TMOD1*, *SLC4A1*, *BCL2L1*, *TRIM58*, *DCAF12*, *NFIX*, *BSG*, *PLVAP*, and *PCSK1N*.

**Table 2:** The results of GO and KEGG pathway enrichment analysis for differentially expressed genes with a trend of up-regulation

Category	Term	Gene symbol	Corrected *P*-value
Gene Ontology term--Biological Process	Porphyrin-containing compound biosynthetic process (GO:0006779)	*SPTB; ANK1*	1.04E-03
Gene Ontology term--Biological Process	Decidualization (GO:0046697)	*BSG; PTGS2*	4.53E-03
Gene Ontology term--Biological Process	Positive regulation of nitric-oxide synthase biosynthetic process (GO:0051770)	*NAMPT; TLR9*	5.34E-03
Gene Ontology term--Biological Process	Positive regulation of NF-kappa B import into nucleus (GO:0042346)	*PTGS2; TLR9*	1.04E-02
Gene Ontology term--Biological Process	Embryo implantation (GO:0007566)	*PTGS2; BSG*	2.92E-02
Gene Ontology term--Biological Process	Adult locomotory behavior (GO:0008344)	*TMOD1; SNCA*	4.20E-02
Gene Ontology term--Cellular Component	Cortical cytoskeleton (GO:0030863)	*TMOD1; SLC4A1; ANK1*	1.88E-04
Gene Ontology term--Cellular Component	Spectrin-associated cytoskeleton (GO:0014731)	*ANK1; SPTB*	1.30E-03
Gene Ontology term--Cellular Component	Basolateral plasma membrane (GO:0016323)	*ANK1; SLC4A1; TLR9*	3.62E-02
Gene Ontology term--Molecular Function	Ankyrin binding (GO:0030506)	*SLC4A1; SPTB*	5.97E-03
Gene Ontology term--Molecular Function	Peroxidase activity (GO:0004601)	*PTGS2; HBD*	6.82E-03
Gene Ontology term--Molecular Function	Structural constituent of cytoskeleton (GO:0005200)	*ANK1; TUBB2A; SPTB*	9.49E-03
Gene Ontology term--Molecular Function	Kinesin binding (GO:0019894)	*SNCA; KLC3*	1.43E-02
KEGG pathway	Small cell lung cancer (ko05222)	*PTGS2; BCL2L1*	3.10E-02
KEGG pathway	NF-kappa B signaling pathway (ko04064)	*PTGS2; BCL2L1*	4.49E-02

### WGCNA

#### Modules identification

**Fig. 1:**
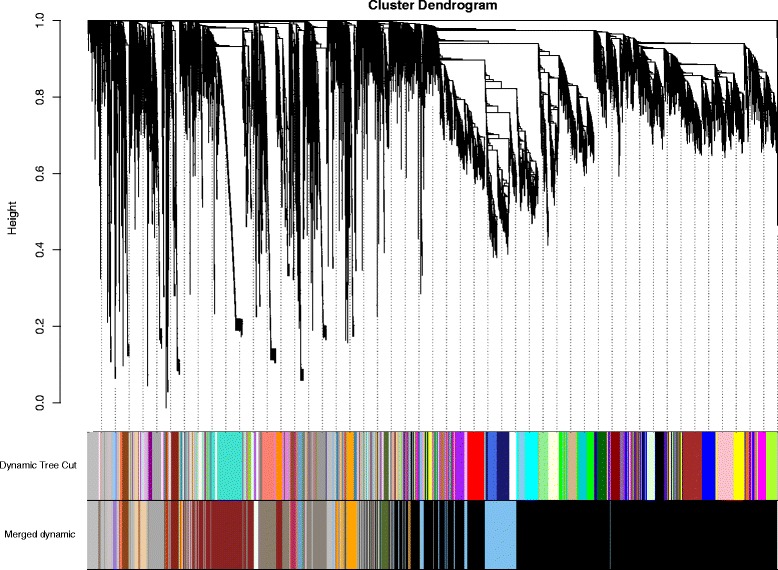
Gene dendrogram obtained by average linkage hierarchical clustering. The color row underneath the dendrogram shows the assigned original module and the merged module

**Fig. 2:**
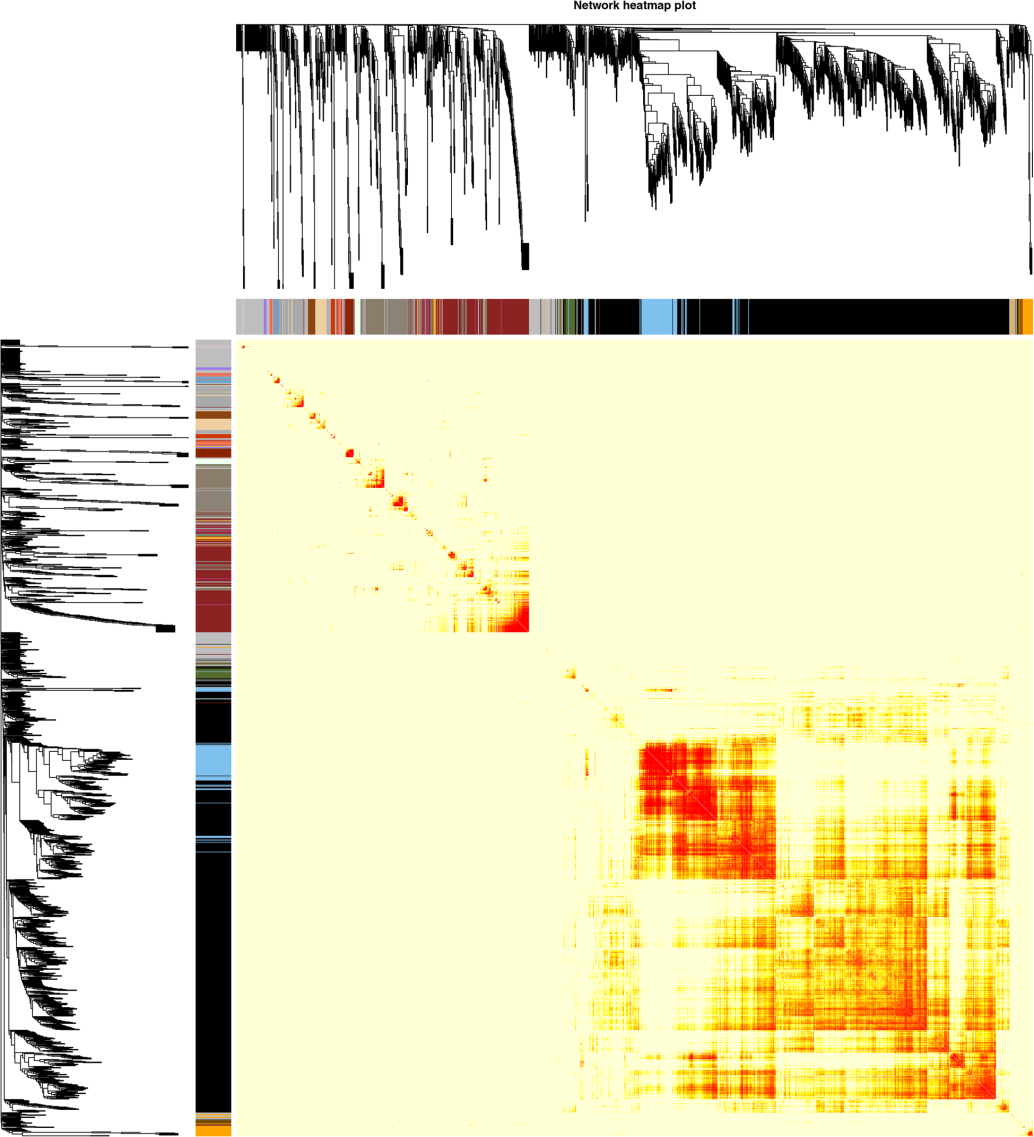
Heatmap plot of topological overlap in the gene network. In the heatmap, each row and column corresponds to a gene, light color denotes low topological overlap, and progressively darker red denotes higher topological overlap. Darker squares along the diagonal correspond to modules. The gene dendrogram and module assignment are shown along the left and top

WGCNA was applied to investigate gene sets that were related to traits of interest-BMI, BMI classes, and disease status using the gene expression data of 7 monozygotic twin pairs. After using a dynamic tree cutting algorithm, a total of 20 distinct co-expression modules containing 48 to 9274 genes per module were identified, and 1912 uncorrelated genes were assigned into a grey module which was ignored in the following study (Fig. 1, and Additional file 3: Table S3). The heatmap plot of topological overlap in the gene network is depicted (Fig. 2).

#### Relating modules to external traits

**Fig. 3:**
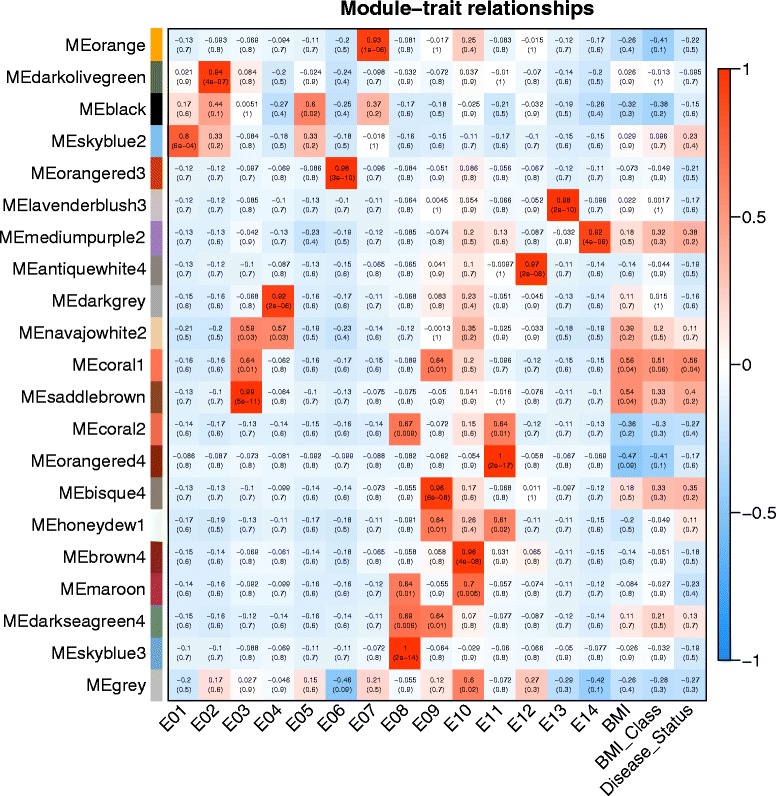
Relationships of consensus module eigengenes and external traits. Each row in the table corresponds to a consensus module, and each column to a sample or trait. Numbers in the table report the correlations of the corresponding module eigengenes and traits, with the *P*-values printed below the correlations in parentheses. The table is color coded by correlation according to the color legend

To understand the physiologic significance of the modules, we correlated the 20 MEs with traits of interest and searched for the most significant associations**.** According to the heatmap of module-trait correlation (Fig. 3), genes clustered in coral1 module (68 genes) had the strongest positive correlation with BMI (*r* = 0.56, *P* = 0.04) and disease status (r = 0.56, *P* = 0.04), whereas statistically nonsignificant correlation was found with BMI classes (*r* = 0.51, *P* = 0.06). Nevertheless, the ME of saddlebrown module (212 genes) was only significantly correlated with BMI (r = 0.56, *P* = 0.04). Thus, we would mainly consider coral1 module in the following because this module may indicate external traits more accurately. None of the other modules had a significant association with external traits.

#### Relationships among modules

**Fig. 4:**
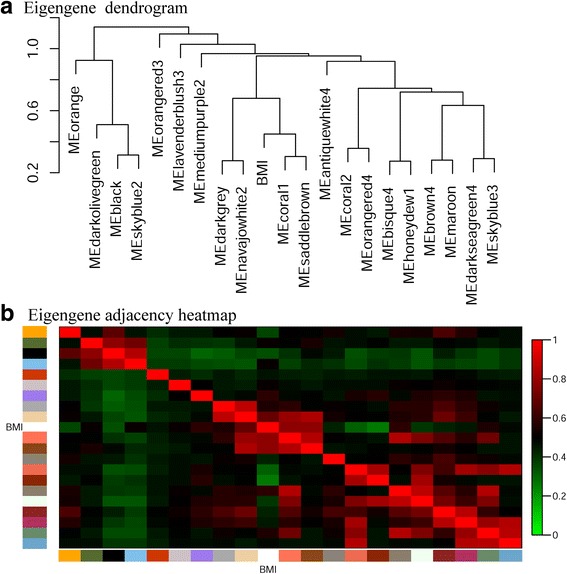
Relationships among modules. **a** Hierarchical clustering of module eigengenes that summarize the modules found in the clustering analysis. Branches of the dendrogram (the meta-modules) group together eigengenes that are positively correlated. **b** Heatmap plot of the adjacencies in the eigengene network including the trait of interest-BMI. Each row and column in the heatmap corresponds to one module eigengene (labeled by color) or BMI. In the heatmap, red represents high adjacency, while blue color represents low adjacency. Squares of red color along the diagonal are the meta-modules

To study the relationships among modules and determine their correlation with trait of BMI, we correlated the MEs. The eigengene network using a dendrogram and a heatmap plot are depicted in Fig. 4. The dendrogram (Fig. 4a) indicated that the coral1 and saddlebrown modules were highly correlated, and trait of BMI fell within the meta-module grouping together the two modules. The heatmap plot (Fig. 4b) showed the detailed eigengenes adjacencies of all modules and trait of BMI.

#### Functional annotation and enrichment analysis for coral1 module

In order to provide an interpretation of the biological mechanism associated with the genes clustered in module of interest--coral1, we conducted functional annotation (Additional file 4: Table S4) and enrichment analysis.

**Fig. 5:**
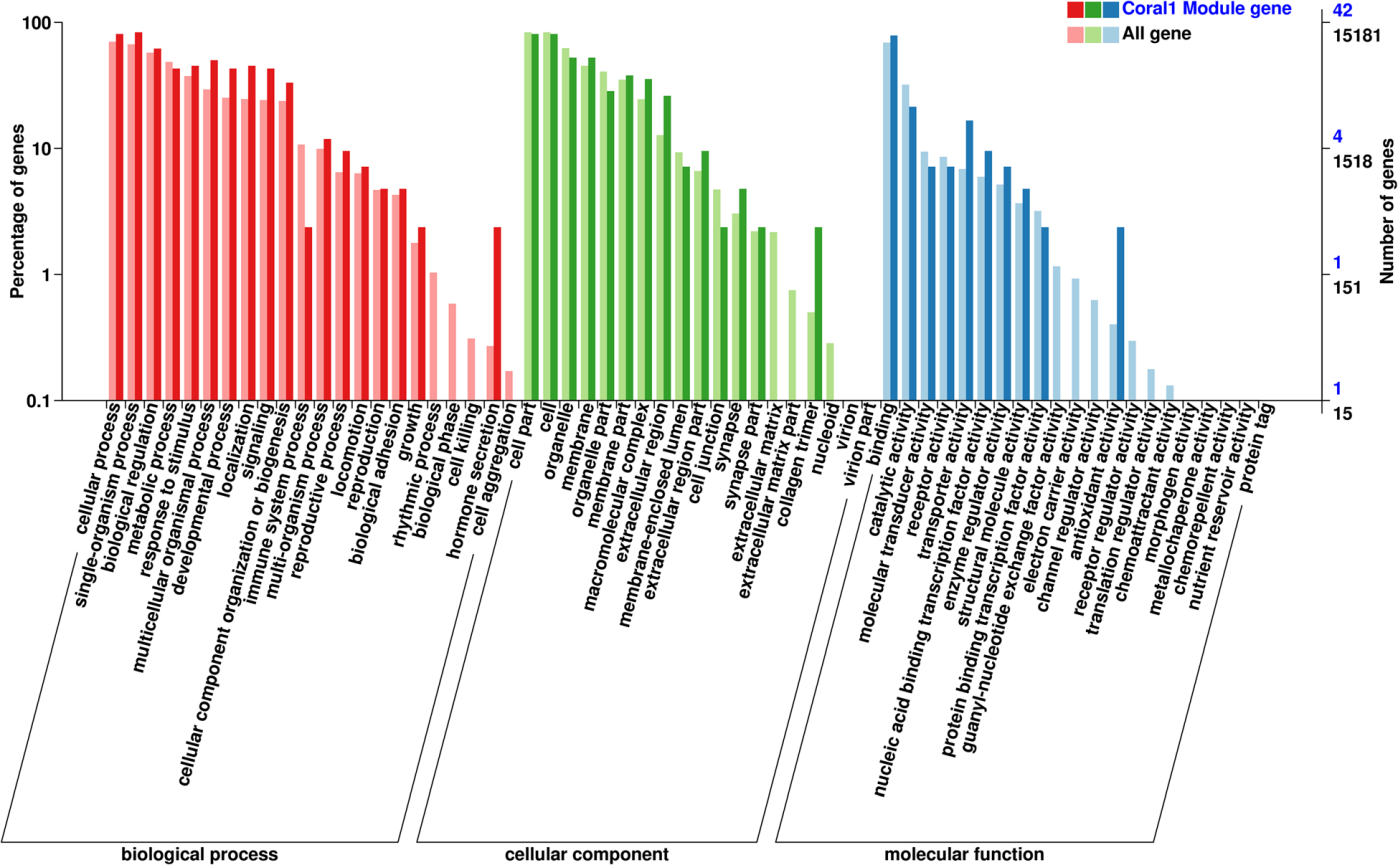
GO classification in coral1 module. Annotation statistics of genes in the secondary node of GO. The horizontal axis shows secondary nodes of three categories in GO. The vertical axis displays the percentage of annotated genes versus the total gene number. The left columns display annotation information of the total genes and the right columns represent annotation information of the genes clustered in coral1 module

Three main annotated categories-biological process, cellular component, and molecular function were obtained in GO database (Fig. 5, and Additional file 5: Table S5). The proportion of genes in coral1 module increased significantly in certain subgroups, including single-organism process (*P* = 0.024), multicellar organismal process (*P* = 0.004), developmental process (*P* = 0.009), localization (*P* = 0.002), signaling (*P* = 0.005), extracellar region (*P* = 0.009), and transporter activity (*P* = 0.023).

As the summarized results of enrichment analysis within GO database shown (Table 3), several potentially important findings emerged (Corrected *P*-value < 0.05). In the biological processes, categories of positive regulation of phospholipase activity (*P* = 2.91E-03), high-density lipoprotein particle clearance (*P* = 4.36E-03), chylomicron remnant clearance (*P* = 4.36E-03), reverse cholesterol transport (*P* = 2.24E-02), and positive regulation of axon extension (*P* = 2.61E-02) were significantly enriched. Among the 6 enrichment categories in the cellular component, intermediate-density lipoprotein particle (*P* = 2.74E-03), chylomicron (*P* = 9.97E-03), low-density lipoprotein particle (*P* = 1.89E-02), and very-low-density lipoprotein particle (*P* = 2.74E-02) were related to lipid transport and metabolism. And categorie of voltage-gated potassium channel complex (*P* = 1.43E-02) may be potentially involved in the regulating energy homeostasis. In the molecular function, the categories of cholesterol transporter activity (*P* = 9.72E-03) and neuropeptide hormone activity (*P* = 1.98E-02) should also be highlighted.

**Table 3:** The results of GO and KEGG pathway enrichment analysis for genes clustered in coral1 module

Category	Term	Gene Symbol	Corrected *P*-value
Gene Ontology term--Biological Process	Positive regulation of phospholipase activity (GO:0010518)	CYR61; APOC2*	2.91E-03
Gene Ontology term--Biological Process	High-density lipoprotein particle clearance (GO:0034384)	APOC2*; APOE*	4.36E-03
Gene Ontology term--Biological Process	Chylomicron remnant clearance (GO:0034382)	APOC2*; APOE*	4.36E-03
Gene Ontology term--Biological Process	Phospholipid efflux (GO:0033700)	APOE*; APOC2*	1.30E-02
Gene Ontology term--Biological Process	Reverse cholesterol transport (GO:0043691)	APOC2*; APOE*	2.24E-02
Gene Ontology term--Biological Process	Positive regulation of axon extension (GO:0045773)	APOE*; RAB25	2.61E-02
Gene Ontology term--Cellular Component	Intermediate-density lipoprotein particle (GO:0034363)	KCNJ1; KCNT1*; KCNA1	2.74E-03
Gene Ontology term--Cellular Component	Chylomicron (GO:0042627)	APOE*; APOC2*	9.97E-03
Gene Ontology term--Cellular Component	Voltage-gated potassium channel complex (GO:0008076)	TBX2*; LBX1*; SOX18*	1.43E-02
Gene Ontology term--Cellular Component	Low-density lipoprotein particle (GO:0034362)	TRH; GAL*	1.89E-02
Gene Ontology term--Cellular Component	Very-low-density lipoprotein particle (GO:0034361)	APOC2*; APOE*	2.74E-02
Gene Ontology term--Cellular Component	Dendrite (GO:0030425)	*MYO1A; NKD2*	3.63E-02
Gene Ontology term--Molecular Function	Cholesterol transporter activity (GO:0017127)	ABCG4*; APOE*	9.72E-03
Gene Ontology term--Molecular Function	Neuropeptide hormone activity (GO:0005184)	GAL*; TRH	1.98E-02
KEGG pathway	Alcoholism (ko05034)	*SHC2; GRIN2C; HIST3H2BB*	1.79E-02
KEGG pathway	Cell adhesion molecules (CAMs) (ko04514)	*SDC1; CADM3; CLDN6*	2.00E-02

Note: * represents the hub genes in coral1 module

As shown in Additional file 6: Table S6, the KEGG annotation results were classified according to KEGG pathway classification. Two pathways of alcoholism and cell adhesion molecules (CAMs) were significantly enriched in KEGG database (Table 3). And the COG function classification results are shown in Additional file 7: Table S7.

#### Hub genes analysis in coral1 module

**Fig. 6:**
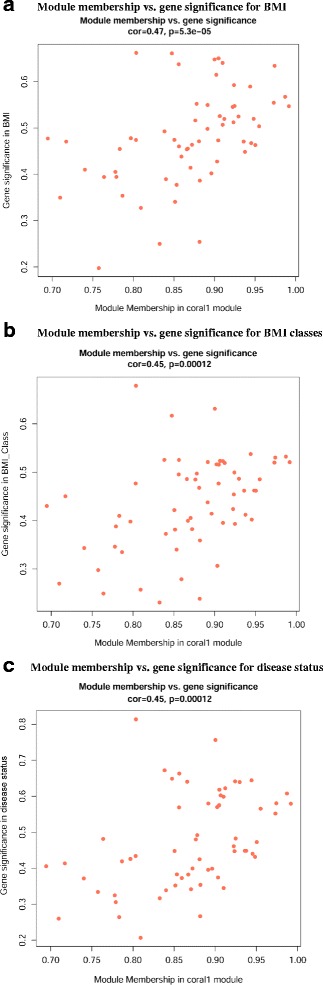
Scatterplots of gene significance (GS) for external traits versus module membership (MM) in the coral1 module. MM and GS for BMI, BMI classes, and disease status exhibit very significant correlations, implying that the most important (central) elements of coral1 module also tend to be highly correlated with these external traits. **a** Module membership vs. gene significance for BMI; (**b**) Module membership vs. gene significance for BMI classes; and (**c**). Module membership vs. gene significance for disease status

**Fig. 7:**
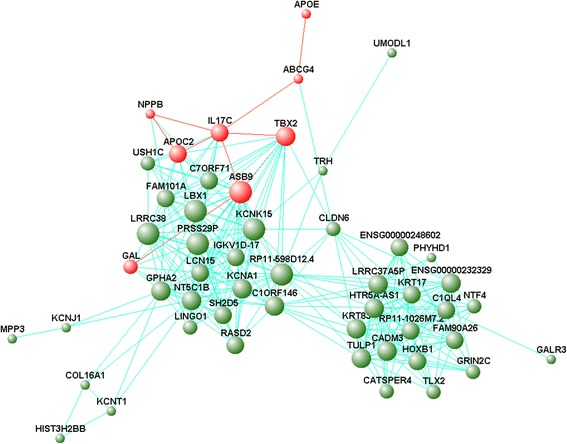
Interaction of gene co-expression patterns by VisANT 5.0 in the coral1 module. The node size and edge number are proportional to degree and connection strength, respectively. Eight red nodes indicate the hub genes potentially related to BMI in the coral1 module. Among the 8 genes, *GAL*, *APOE*, *APOC2*, and *NPPB* have been demonstrated to be associated with obesity and the others would be associated with obesity as the related works suggested

Figure 6 shows the scatterplots of GS for traits of BMI, BMI classes, and disease status versus MM in coral1 module. MM and GS for BMI (Fig. 6a), BMI classes (Fig. 6b), and disease status (Fig. 6c) exhibited very significant positive correlations, implying that the most important (central) elements of coral1 module also tended to be highly correlated with these external traits. The identified 21 hub genes (Additional file 8: Table S8) included *GAL*, *ASB9*, *KCNT1*, *NPPB*, *TBX2*, *KCNK15*, *IL17C*, *APOE*, *LBX1*, *LRRC38*, *LINGO1*, *ABCG4*, *LCN15*, *RFLNA*, *SOX18*, *C1orf146*, *APOC2*, *PRSS29P*, *LOC102724223*, *C7orf71*, and *IGKV1D-17*. The visualized plot of the gene-gene interaction network in coral1 module is shown in Fig. 7.

The 21 hub genes were involved in several enriched functional items (Table 3), including high-density lipoprotein particle clearance, chylomicron remnant clearance, phospholipid efflux, reverse cholesterol transport, chylomicron, voltage-gated potassium channel complex, very-low-density lipoprotein particle, and cholesterol transporter activity, most of which were associated with lipid transport and metabolism. None of the 68 genes in coral1 module was identified as DEGs.

#### Saddlebrown module

In the functional annotation analysis within GO database, the proportion of genes in saddlebrown module increased in subgroups of extracellular region (*P* = 0.002) and extracellular region part (*P* = 0.019) (Additional file 9: Figure S1, and Additional file 10: Table S9). The categories of structural constituent of eye lens (*P* = 1.14E-02) and troponin T binding (*P* = 2.75E-02) were significantly enriched in the molecular function, whereas no categories were significantly enriched in biological process and cellular component. The results of KEGG pathway classification and COG function classification are shown in Additional file 11: Table S10 and Additional file 12: Table S11, respectively. BMI based GS and MM exhibited a very significant correlation in saddlebrown module (Additional file 13: Figure S2), and hub genes of *KCNN1*, *CNN1*, and *AQP10* were identified as DEGs.

## Discussion

In the differential expression analysis based on the gene expression data of 7 BMI-discordant monozygotic twin pairs, we identified 32 genes with a trend of up-regulation in twins with higher BMI when compared to their siblings. Several potentially important enrichment findings emerged, including positive regulation of nitric-oxide synthase biosynthetic process, positive regulation of NF-kappa B import into nucleus, peroxidase activity, and NF-kappa B signaling pathway (Table 2). And up-regulated genes-*NAMPT*, *TLR9*, *PTGS2*, *HBD*, and *PCSK1N* might be associated with obesity risk. In addition, we also applied WGCNA to quantitatively analyze the interconnectedness of gene expression data and assessed the importance of genes within the networks. Among the 20 distinct co-expression modules identified, genes clustered in coral1 module had the strongest positive correlation with BMI and disease status **(**Fig. 3), indicating that the highly co-expressed genes in this module had potential biological significance. Functional enrichment analysis revealed several significant enrichments of BMI-related categories for coral1 module. Importantly, several hub genes were strongly related to lipid transport and metabolism (Table 3) and may be particularly valuable for identifying the candidate biomarkers and therapeutic targets for obesity assessed by BMI. Besides, the ME of saddlebrown module was also significantly correlated with BMI (Fig. 3) and 3 hub genes were identified as DEGs.

Obesity is a complex disease under the control of both genetic and environmental factors through the interface of epigenetics, where different combinations of genetic and epigenetic variations can lead to a common phenotype. Considering this, we would not necessarily expect each of the 7 BMI-discordant monozygotic twin pairs to present exactly the same series of gene aberrations, and the stringency of the criterion on the commonality of gene changes was relaxed. In the differential expression analysis, it appeared that 32 genes were with a trend of up-regulation in at least three of twins with higher BMI when compared to their siblings (Additional file 2: Table S2). Besides, four potentially important enrichment findings emerged and 5 up-regulated genes associated with obesity risk were identified as follows (Table 2).

Nitric oxide (NO), whose production is mostly through the action of the nitric oxide synthase (NOS) family of enzymes, is emerging as a central regulator of energy metabolism and body composition. The isoform of inducible nitric oxide synthase (iNOS)-derived NO can promote insulin resistance and inflammation in key peripheral tissues such as liver, skeletal muscle, and adipose tissue. In addition, iNOS may affect glucose homeostasis. Thus, the iNOS isoform appears to promote deleterious changes in metabolism [53]. Considering this, the two up-regulated genes (*NAMPT* and *TRL9*) involved in the category of positive regulation of nitric-oxide synthase biosynthetic process should be considered notably (Table 2). And it is indicated that the protein of *NAMPT* gene can directly activate pathways leading to iNOS induction [54].

It has been revealed that a characteristic feature of obesity linking it to insulin resistance is the presence of chronic low-grade inflammation which is indicative of activation of the innate immune system. The IKK/NF-κB pathway is a well-known inflammatory signaling pathway involved in the pathogenesis of obesity [55, 56], and the two genes-*PTGS2* and *TLR9* involved in this enrichment term should also be focused (Table 2). In addition, the protein encoded by *PTGS2* gene is indicated to be linked with energy homeostasis and metabolic processes based on a cohort of children presenting with syndromic obesity [57]. Even though both these two genes were enriched to the NF-kappa B signaling pathway in KEGG database (Table 2), *PTGS2* gene was involved in the inflammation process while *BCL2L1* gene might be related to survival process.

In mammals, the peroxidases comprise 8 glutathione peroxidases (GPx1–GPx8) so far identified. Too much data regarding the association between obesity and GPx1, GPx3, GPx4, and GPx7 has been reported [58]. Thus, the two genes of *PTGS2* and *HBD* could be regard as the candidates for further research (Table 2).

Moreover, SNPs in or near *PCSK1* loci may also contribute to obesity risk [59, 60]. The associations with BMI for other DEGs should be explored further.

To validate the identified DEGs further, we compared previously implicated BMI-related gene expression differences [52] with ours. This comparison revealed consistency for positive BMI-associated expression differences including *HBD*, *XK*, *SELENBP1*, *SNCA*, *LAS2*, *PLEK2*, *GLRX5*, *TMOD1*, *SLC4A1*, *BCL2L1*, *TRIM58*, *DCAF12*, *NFIX*, *BSG*, *PLVAP*, and *PCSK1N*. Two consistent genes (*SNCA* and *DCAF12*) were also revealed when compared with the BMI-related genes by the Data-driven Expression Prioritized Integration for Complex Traits (DEPICT) method.

In the WGCNA, the proportion of genes in coral1 module increased significantly in subgroups of developmental process, signaling, extracellar region, and transporter activity (Fig. 5, and Additional file 5: Table S5), indicating that these functions may be associated with metabolism and accelerated growth and development of obesity individuals. Notably, GO enrichment analysis also provided more significant results with more biological meanings as follows (Table 3).

Obese subjects frequently suffer atherogenic dyslipidemia which is commonly manifested as elevated plasma free fatty acids, triglycerides (TG) and very low-density lipoprotein (VLDL) levels, decreased high-density lipoprotein cholesterol (HDL-C) levels, and abnormal low-density lipoprotein cholesterol (LDL-C) [61, 62]. Our study suggested that categories involved in lipid metabolism and transport were significantly enriched within the coral1 module, including high-density lipoprotein particle clearance, reverse cholesterol transport, low-density lipoprotein particle, cholesterol transporter activity, chylomicron, chylomicron remnant clearance, very-low-density lipoprotein particle, and intermediate-density lipoprotein particle (Table 3).

Category of positive regulation of phospholipase activity was also enriched in the coral1 module (Table 3). Phospholipids were identified as potential biomarkers for obesity [63]. And it was reported that members of the phospholipase A2 (PLA2) family of enzymes, such as PLA2G1B [64], PLA2G5, and PLA2G2E [65], can serve a distinct role in generating active lipid metabolites, which can promote inflammatory metabolic diseases including obesity [66, 67]. In addition, AdPLA enzyme in white adipose tissue can function as a regulator of lipolysis through increasing prostaglandin E2 (PGE2) formation and decreasing intracellular cAMP [68].

The hypothalamic peptides, such as neuropeptide Y (NPY) and melanin concentrating hormone (MCH) [69], and the peripheral neuropeptides, such as hormone leptin [70], play important roles in regulating food intake and maintaining energy balance [71]. Normally, a dynamic equilibrium exists between orexigenic peptides and anorexigenic peptides [70]. And after receiving stimulus information of neural signal, hormone signal, and metabolites, etc., the hypothalamus appestat maintains the dynamic equilibrium of energy by neuro-humoral response. Therefore, the enriched category of neuropeptide hormone activity may also exert a significant meaning regarding obesity (Table 3).

Another significant GO enriched category was voltage-gated potassium channel (Kv) complex (Table 3). Studies had suggested the relation of subtype-Kv1.3 to insulin sensitivity and the participation of Kv1.3 in regulating energy homeostasis and body weight [72, 73]. Hence, Kv1.3 may be a putative and promising pharmacological target for the treatment of obesity, type II diabetes mellitus and related metabolic diseases [72].

Two pathways of alcoholism and cell adhesion molecules (CAMs) were significantly enriched in KEGG database (Table 3). A growing body of literatures indicate that overlapping central pathways may be involved with uncontrolled eating and excessive ethanol drinking [74]. And emerging link between familial alcoholism risk and obesity in women and possibly in men is identified in recent years [75]. Furthermore, some genetic variants are associated with both alcohol dependence and obesity [76, 77]. Therefore, the genes involved in the alcoholism pathway may be used as potential links between alcoholism and obesity, and as promising targets for controlling ethanol abuse and food intake. As for the CAMs, a review concluded that anthropometric indicators, body composition and eating pattern positively modulate the subclinical inflammation of obesity through reducing CAMs and chemokines [78]. Moreover, a recent study also identified the relationship of adiposity to several CAMs [79].

We visualized the gene-gene interaction network in coral1 module to obtain an insight on the hidden mechanisms (Fig. 7), and a total of 21 hub genes were identified (Additional file 8: Table S8). The hub genes were involved in various gene families and might serve as candidates for additional mechanistic studies and therapeutic interventions. Hub genes of *GAL*, *APOE*, *APOC2*, and *NPPB* have been demonstrated to be associated with obesity as follows: (I) *GAL*: Galanin peptides, as the protein for *GAL*, is undoubtedly involved in the regulation of food intake and body weight. It has been identified that both central galanin and peripheral galanin can affect appetite, food intake and body weight of animals, and the latter can also affect gastrointestinal motility and brown adipose tissue activity [80, 81]. Particularly, newly discovered galanin-like peptide (GALP) may play a role in boosting appetite, body weight, and obesity [80, 82]. Overall, galanin and its receptors may serve as a novel anti-obesity strategy in the future. (II) *APOE*: ApoE synthesized by adipocyte is a polymorphic glycoprotein in humans, and is a major constituent of HDL, VLDL, and remnant lipoproteins (RLPs). ApoE was identified playing an important role in the development of obesity and insulin resistance in experimental mouse models [83], and the mutation in *APOE* was involved in lipid metabolism [84] and lipid levels [85] in population studies. Moreover, an equally vital role in adipocyte triglyceride accumulation and VLDL-induced adipogenesis was summarized [86]. Overall, it has been identified that *APOE* expression serves as a key peripheral contributor to the development of obesity and related metabolic dysfunctions [83, 87]. (III) *APOC2*: Apolipoprotein C-II (ApoC-II), as a constituent of chylomicrons, VLDL, LDL and HDL, is a cofactor for lipoprotein lipase, which can hydrolyze TG. The gene *APOC2* mutation can result in hypertriglyceridemia, which is one of the main characteristics of obese subjects [88]. Besides, an excess of ApoC-II is related to increase of triglyceride-rich particles and alterations in HDL particle distribution [89]. (IV) *NPPB*: A growing body of evidence indicates that the natriuretic peptides (NPs) system holds the potential to be amenable to therapeutical intervention against obesity. Vila, G, et al. demonstrate that B-Type Natriuretic Peptide (BNP) plays an important role in reducing circulating ghrelin concentrations, decreasing hunger, and increasing feeling of satiety in healthy individuals [90]. Moreover, the function of enhancing lipolysis and energy expenditure, and modulating adipokine release and food intake is also identified [91]. In addition, one recent review emphasized the ability of NPs to regulate body weight and energy homeostasis by driving lipolysis, facilitating beiging of adipose tissues, and promoting lipid oxidation and mitochondrial respiration [92]. Moreover, another review drew the similar conclusion [93].

Although there was no strong indication that *TBX2*, *ASB9*, *IL17C*, and *ABCG4* were the causal variant of obesity in the population, studies showed that these genes may also be part of the multifactorial etiology of this complex condition as follows: (I) *TBX2*: The results of a prospective cohort on the associations of menarche-related genetic variants with pubertal growth in adolescents indicated that SNPs (rs757608) near *TBX2* is associated with a rapid weight gain [94]. (II) *ASB9*: It indicated that overexpression of *ASB9* can induce ubiquitination of ubiquitous mitochondrial creatine kinase (uMtCK) [95] in a specific, SOCS box-independent manner [96]. The intracellular creatine kinase (CK) system may be involved in the storage of fat and the development of obesity [97–99]. Besides, one cross-sectional study recently provided further evidence that CK may play a role in the pathophysiology of obesity and serve as a marker to identify individuals at risk for obesity [100]. (III) *IL17C*: Obesity, in some sense, is considered to be an inflammatory predisposition. And interleukin-17 (IL-17) may impact adipose tissue due to the association with induction of tissue inflammation. Particularly, the potential implications of IL-17 in relation to obesity has been consolidated by Ahmed, M and Gaffen, SL [101]. And one study also suggested a linear negative association between IL-17 and visceral adipose tissue thickness [102]. However, the exact role of IL-17C in obesity remains to be explored. (IV) *ABCG4*: An additional hub gene that should be further investigated is *ABCG4*, one member of the *ABCG* family. Studies indicated that ABCG4 promotes cholesterol efflux from cells to HDL [103, 104].

Even though no sufficient studies showed the association of two genes of *KCNT1* and *LBX1* with obesity, the results of functional annotation and enrichment analysis indicated that they were involved in intermediate-density lipoprotein particle and voltage-gated potassium channel complex, respectively. Thus, they may also be regarded as the targets for etiology research of obesity. Other hub genes in coral1 module were of unknown function in terms of obesity currently, whereas they may also be interesting potential candidates to be future researched and validated.

Among the hub genes in saddlebrown module, *KCNN1*, *CNN1*, and *AQP10* were up-regulated with increasing BMI in twins. (I) *KCNN1*: The lipotoxicity in morbid obesity can gradually impair insulin action in the liver and muscle, aggravating insulin resistance [105], and Ye, J proposed an energy-based concept of insulin resistance, in which insulin resistance is a result of energy surplus in cells [106]. The protein of gene *KCNN1*--small conductance calcium-activated potassium channel protein 1, can serve as a key regulator of excitability and endocrine function in beta cells [107]. (II) *AQP10*: Aquaglyceroporins, such as AQP10, represent novel additional pathways for the transport of glycerol in human adipocytes [108], and the deregulation in the expression of aquaglyceroporins in adipose tissue is associated with human obesity [109, 110].

Several strengths must be noticed in our study. First, gene expression levels may be under the effect of subjects’ genetic background, gender, age, and environmental exposures as well as by some experimental variables related to clinical sampling, processing, and data analysis. However, the discordant monozygotic model, which is characteristic of the genetic similarity and rearing-environment sharing, is becoming a popular and powerful tool for identifying non-genetic contributions to a phenotype variation including difference in gene expression. Hence, our results of WGCNA, based on the expression data generated from BMI-discordant monozygotic model, may be more credible. Another strength of our study was that the WGCNA provides information on the correlated patterns of expression and the effect of networks of genes, which is useful for detecting important biological pathways or gene-gene interactions related to obesity. Specifically, a set of genes sharing similar functions and correlated to one another in coral1 and saddlebrown modules were identified in our study, some of which have already been verified to play efficient roles in obesity.

Nevertheless, our study has potential limitations as well. First, our study was with small sample size and limited statistical power resulting from the challenges of identifying and recruiting qualified monozygotic twins discordant for BMI. The BMI-discordant monozygotic model, however, helps to mitigate confounding factors associated with genetic polymorphisms in studies of unrelated human subjects and to identify non-genetic contributions to a phenotype variation including difference in gene expression. Besides, we had identified significant genes and specific modules potentially related to BMI. It’s still important and necessary to validate our findings when more sample size is acquired. Second, we couldn’t validate our results with an external and independent dataset because of lacking public BMI-discordant monozygotic dataset with adequate size currently. However, we compared previously implicated BMI-related gene expression differences with our findings, and 16 consistent positive BMI-associated findings were revealed. Third, some genes may be involved in multiple processes/functions which require different gene sets. However, it was difficult to characterize such gene interactions because of the impossibility of forming overlapping modules by WGCNA. Fourth, as in any other studies based on microarray technology, changes in protein levels may not reflect similar changes in mRNA levels accurately because post-translational modification also acts importantly in controlling biological processes. Hence, it may be necessary to validate our results by other techniques. And fifth, in saddlebrown module, the 3 hub genes also identified as DEGs were differentially expressed in just one twin pair. More studies are needed to confirm these results.

## Conclusions

In summary, we identified 32 DEGs with a trend of up-regulation in twins with higher BMI when compared to their siblings in the differential expression analysis and determined one module most positively correlated with BMI and several hub genes in the WGCNA. The potentially significant genes and pathways correlated with BMI identified in our analysis may help further elucidate the molecular mechanisms underlying obesity development and provide novel insights regarding future prognostic and therapeutic approaches. Further analysis and validation of the candidate biomarkers of obesity reported here are necessary, including those that have not yet been definitely identified.

## Additional files

Additional file 1: Table S1. Summary of the sequencing reads and the mapped results for the 7 monozygotic twin pairs (DOCX 17 kb)

Additional file 2: Table S2. The summary of differentially expressed genes with a trend of up-regulation in BMI-discordant monozygotic twin pairs and the corresponding functional annotation results (XLSX 23 kb)

Additional file 3: Table S3. Module assignments for genes in network of WGCNA (XLSX 302 kb)

Additional file 4: Table S4. The result for functional annotation within databases of NR, COG, KOG, KEGG, and GO for genes clustered in coral1 module (XLSX 24 kb)

Additional file 5: Table S5. GO classification of genes in coral1 module (XLSX 13 kb)

Additional file 6: Table S6. KEGG categories of genes in coral1 module (XLSX 12 kb)

Additional file 7: Table S7. COG function classification in coral1 module (XLSX 12 kb)

Additional file 8: Table S8. The hub genes found by the criterion of BMI based GS > 0.2 and MM > 0.8 with a threshold of *P*-value <0.05 in coral1 module (DOCX 16 kb)

Additional file 9: Figure S1. GO classification in saddlebrown module. Annotation statistics of genes in the secondary node of GO. The horizontal axis shows secondary nodes of three categories in GO. The vertical axis displays the percentage of annotated genes versus the total gene number. The left columns display annotation information of the total genes and the right columns represent annotation information of the genes clustered in saddlebrown module. (TIFF 777 kb)

Additional file 10: Table S9. GO classification of genes in saddlebrown module (XLSX 14 kb)

Additional file 11: Table S10. KEGG categories of genes in saddlebrown module (XLSX 18 kb)

Additional file 12: Table S11. COG function classification in saddlebrown module (XLSX 15 kb)

Additional file 13: Figure S2. Scatterplots of BMI based gene significance (GS) versus module membership (MM) in the saddlebrown module. GS for BMI and MM exhibit a very significant correlation, implying that the most important (central) elements of saddlebrown module also tend to be highly correlated with BMI trait (TIFF 168 kb)

## Abbreviations

BMI: Body mass index
DEGs: Differentially expressed genes
FC: Fold change
FDR: False discovery rate
GO: Gene Ontology
GS: Gene significance
KEGG: Kyoto Encyclopedia of Genes and Genomes
MM: Module membership
WGCNA: Weight gene co-expression network analysis
