# Supplementary material for: Weighted gene co-expression network analysis of expression data of monozygotic twins identifies specific modules and hub genes related to BMI
Source: BMC Genomics. 2017 Nov 13;18:872. doi: 10.1186/s12864-017-4257-6 (PMC5683603; doi:10.1186/s12864-017-4257-6)
Supplement: Supplementary file 1 — Summary of the sequencing reads and the mapped results for the 7 monozygotic twin pairs (DOCX 17 kb) [file 12864_2017_4257_MOESM1_ESM.docx]

**Additional file 1 Table S1**. Summary of the sequencing reads and the mapped results for the 7 monozygotic twin pairs

| **Subject ID** | **Raw reads** | **Clean reads** | **Clean bases** | **GC Content** | **%≥Q30** | **Mapped Reads** | **Uniq Mapped Reads** | **Multiple Map Reads** | **Reads Map to '+'** | **Reads Map to '-'** |
| --- | --- | --- | --- | --- | --- | --- | --- | --- | --- | --- |
| E01 | 20,235,588 | 13,175,659 | 671,884,265 | 57.88% | 92.26% | 11,544,510 (87.62%) | 8,409,876 (63.83%) | 3,134,634 (23.79%) | 5,637,578 (42.79%) | 5,824,340 (44.21%) |
| E02 | 16,409,091 | 11,924,297 | 608,079,545 | 57.69% | 94.16% | 11,025,755 (92.46%) | 7,627,586 (63.97%) | 3,398,169 (28.50%) | 5,418,454 (45.44%) | 5,546,485 (46.51%) |
| E03 | 17,998,847 | 11,908,656 | 607,278,810 | 60.78% | 92.98% | 10,912,268 (91.63%) | 6,018,781 (50.54%) | 4,893,487 (41.09%) | 5,308,111 (44.57%) | 5,569,082 (46.76%) |
| E04 | 18,666,841 | 12,414,080 | 633,068,390 | 62.26% | 92.60% | 11,396,763 (91.81%) | 5,308,580 (42.76%) | 6,088,183 (49.04%) | 5,505,924 (44.35%) | 5,858,765 (47.19%) |
| E05 | 15,204,595 | 11,962,437 | 610,015,273 | 57.31% | 94.02% | 11,091,166 (92.72%) | 8,572,887 (71.67%) | 2,518,279 (21.05%) | 5,462,768 (45.67%) | 5,551,336 (46.41%) |
| E06 | 22,052,642 | 14,285,308 | 728,502,701 | 61.36% | 92.49% | 12,757,580 (89.31%) | 6,279,868 (43.96%) | 6,477,712 (45.35%) | 6,247,883 (43.74%) | 6,492,521 (45.45%) |
| E07 | 16,860,495 | 11,553,854 | 589,192,308 | 58.62% | 94.29% | 10,502,038 (90.90%) | 6,500,524 (56.26%) | 4,001,514 (34.63%) | 5,112,391 (44.25%) | 5,325,323 (46.09%) |
| E08 | 17,772,171 | 11,824,318 | 602,991,670 | 60.88% | 93.88% | 10,938,193 (92.51%) | 5,494,359 (46.47%) | 5,443,834 (46.04%) | 5,263,733 (44.52%) | 5,642,667 (47.72%) |
| E09 | 18,649,833 | 12,330,870 | 628,819,526 | 60.02% | 93.90% | 11,292,200 (91.58%) | 6,055,037 (49.10%) | 5,237,163 (42.47%) | 5,444,416 (44.15%) | 5,796,542 (47.01%) |
| E10 | 17,060,751 | 12,029,979 | 613,462,183 | 60.01% | 93.57% | 10,888,684 (90.51%) | 6,032,520 (50.15%) | 4,856,164 (40.37%) | 5,224,387 (43.43%) | 5,593,222 (46.49%) |
| E11 | 18,853,362 | 12,537,011 | 639,333,382 | 61.42% | 93.31% | 11,511,421 (91.82%) | 5,678,068 (45.29%) | 5,833,353 (46.53%) | 5,498,063 (43.85%) | 5,979,478 (47.69%) |
| E12 | 20,187,838 | 13,045,821 | 665,282,045 | 59.83% | 92.41% | 11,540,848 (88.46%) | 6,479,401 (49.67%) | 5,061,447 (38.80%) | 5,584,426 (42.81%) | 5,889,504 (45.14%) |
| E13 | 20,595,652 | 13,359,644 | 681,283,798 | 61.12% | 92.61% | 12,097,095 (90.55%) | 6,017,078 (45.04%) | 6,080,017 (45.51%) | 5,851,731 (43.80%) | 6,214,624 (46.52%) |
| E14 | 17,874,341 | 11,587,153 | 590,895,803 | 61.11% | 93.90% | 10,797,229 (93.18%) | 5,287,350 (45.63%) | 5,509,879 (47.55%) | 5,248,572 (45.30%) | 5,532,508 (47.75%) |

Note: GC content: clean data G and C percentage of the total bases; Q30: quality score of base is greater than or equal to 30% of the total bases; Mapped reads: the number of reads mapped to reference genome and the percentage of the total clean reads; Uniq mapped reads: the number of reads mapped to one location of reference genome and the percentage of the total clean reads; Multiple mapped reads: the number of reads mapped to multiple locations of reference genome and the percentage of the total clean reads; Reads map to '+': the number of reads mapped to plus-strands of reference genome and the percentage of the total clean reads; Reads map to '-': the number of reads mapped to minus-strands of reference genome and the percentage of the total clean reads.
