# Supplementary material for: Weighted gene co-expression network analysis of expression data of monozygotic twins identifies specific modules and hub genes related to BMI
Source: BMC Genomics. 2017 Nov 13;18:872. doi: 10.1186/s12864-017-4257-6 (PMC5683603; doi:10.1186/s12864-017-4257-6)
Supplement: Supplementary file 8 — The hub genes found by the criterion of BMI based GS > 0.2 and MM > 0.8 with a threshold of P-value <0.05 in coral1 module (DOCX 16 kb) [file 12864_2017_4257_MOESM8_ESM.docx]

**Additional file 8: Table S8**. The hub genes found by the criterion of BMI based GS>0.2 and MM>0.8 with a threshold of *P*-value < 0.05 in coral1 module

| **Gene ID** | **Gene symbol** | **Gene full name** | **MM** | ***P*-MM** | **GS** | ***P*-GS** |
| --- | --- | --- | --- | --- | --- | --- |
| ENSG00000069482 | *GAL* | Galanin and GMAP prepropeptide | 0.803548244 | 5.36E-04 | 0.662283933 | 9.86E-03 |
| ENSG00000102048 | *ASB9* | Ankyrin repeat and SOCS box containing 9 | 0.944058885 | 3.92E-07 | 0.589472603 | 2.65E-02 |
| ENSG00000107147 | *KCNT1* | Potassium sodium-activated channel subfamily T member 1 | 0.878041467 | 3.63E-05 | 0.552159482 | 4.06E-02 |
| ENSG00000120937 | *NPPB* | Natriuretic peptides B | 0.85592234 | 9.40E-05 | 0.637766872 | 1.41E-02 |
| ENSG00000121068 | *TBX2* | T-box 2 | 0.974073764 | 4.15E-09 | 0.634283311 | 1.48E-02 |
| ENSG00000124249 | *KCNK15* | Potassium two pore domain channel subfamily K member 15 | 0.987094396 | 6.49E-11 | 0.567429283 | 3.43E-02 |
| ENSG00000124391 | *IL17C* | Interleukin 17C | 0.924056852 | 2.35E-06 | 0.592613282 | 2.55E-02 |
| ENSG00000130203 | *APOE* | Apolipoprotein E | 0.92471899 | 2.23E-06 | 0.547486725 | 4.27E-02 |
| ENSG00000138136 | *LBX1* | Ladybird homeobox 1 | 0.944058885 | 3.92E-07 | 0.589472603 | 2.65E-02 |
| ENSG00000162494 | *LRRC38* | Leucine rich repeat containing 38 | 0.944058885 | 3.92E-07 | 0.589472603 | 2.65E-02 |
| ENSG00000169783 | *LINGO1* | Leucine rich repeat and Ig domain containing 1 | 0.891561071 | 1.85E-05 | 0.549715415 | 4.17E-02 |
| ENSG00000172350 | *ABCG4* | ATP binding cassette subfamily G member 4 | 0.922446129 | 2.65E-06 | 0.545505247 | 4.36E-02 |
| ENSG00000177984 | *LCN15* | Lipocalin 15 | 0.900204906 | 1.15E-05 | 0.648112644 | 1.22E-02 |
| ENSG00000178882 | *RFLNA* | Refilin A | 0.909975502 | 6.31E-06 | 0.640657747 | 1.36E-02 |
| ENSG00000196364 | *PRSS29P* | Protease, Serine 29, Pseudogene | 0.944058885 | 3.92E-07 | 0.589472603 | 2.65E-02 |
| ENSG00000198555 | *LOC102724223* | Sodium-and chloride-dependent creatine transporter 1-like | 0.991771786 | 4.40E-12 | 0.547072577 | 4.29E-02 |
| ENSG00000203883 | *SOX18* | SRY-box 18 | 0.847526297 | 1.30E-04 | 0.661301337 | 1.00E-02 |
| ENSG00000203910 | *C1orf146* | Chromosome 1 open reading frame 146 | 0.97306723 | 5.20E-09 | 0.554609665 | 3.96E-02 |
| ENSG00000222004 | *C7orf71* | Chromosome 7 open reading frame 71 | 0.904977255 | 8.64E-06 | 0.650530034 | 1.18E-02 |
| ENSG00000234906 | *APOC2* | Apolipoprotein C2 | 0.904977255 | 8.64E-06 | 0.650530034 | 1.18E-02 |
| ENSG00000242766 | *IGKV1D-17* | Immunoglobulin kappa variable 1D-17 | 0.902326125 | 1.01E-05 | 0.615000235 | 1.92E-02 |

**Note**: GS: gene significance; MM: module membership.
