# Supplementary figures and images for: Weighted gene co-expression network analysis of expression data of monozygotic twins identifies specific modules and hub genes related to BMI
Source: BMC Genomics. 2017 Nov 13;18:872. doi: 10.1186/s12864-017-4257-6 (PMC5683603; doi:10.1186/s12864-017-4257-6)

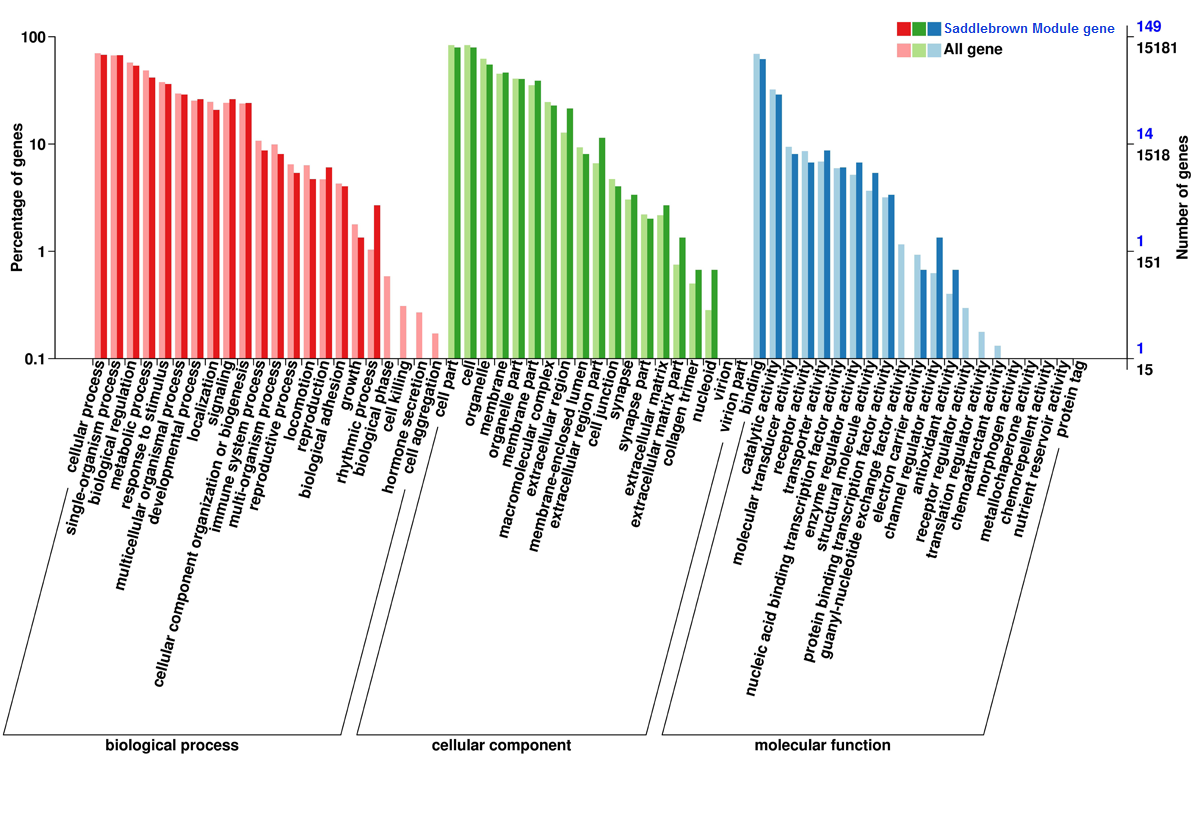

Supplement: Supplementary file 9 — GO classification in saddlebrown module. Annotation statistics of genes in the secondary node of GO. The horizontal axis shows secondary nodes of three categories in GO. The vertical axis displays the percentage of annotated genes versus the total gene number. The left columns display annotation information of the total genes and the right columns represent annotation information of the genes clustered in saddlebrown module. (TIFF 777 kb) [file 12864_2017_4257_MOESM9_ESM.tif]

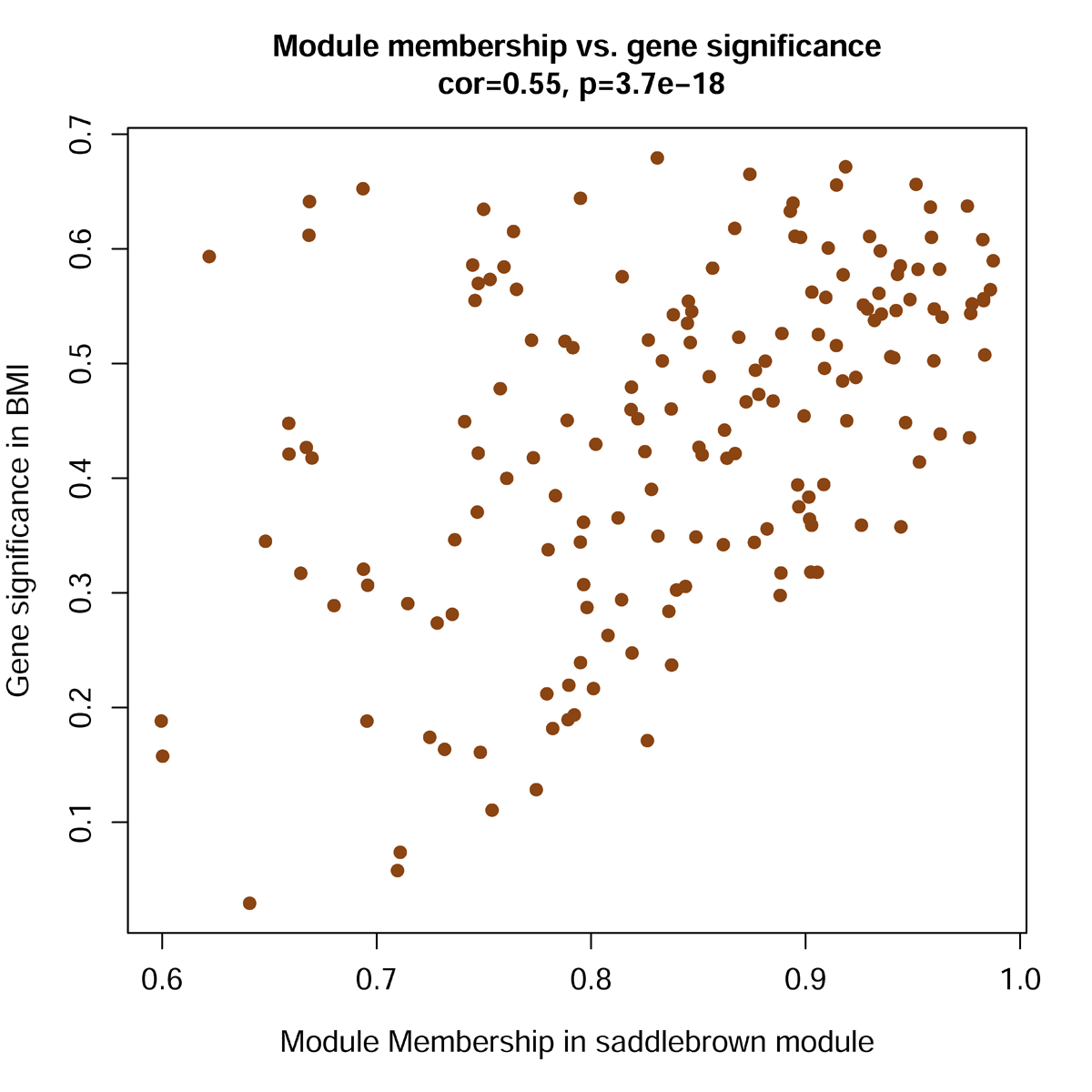

Supplement: Supplementary file 13 — Scatterplots of BMI based gene significance (GS) versus module membership (MM) in the saddlebrown module. GS for BMI and MM exhibit a very significant correlation, implying that the most important (central) elements of saddlebrown module also tend to be highly correlated with BMI trait (TIFF 168 kb) [file 12864_2017_4257_MOESM13_ESM.tif]
